# LncRNA DILC participates in rheumatoid arthritis by inducing apoptosis of fibroblast-like synoviocytes and down-regulating IL-6

**DOI:** 10.1042/BSR20182374

**Published:** 2019-05-02

**Authors:** Guan Wang, Lian Tang, Xihai Zhang, Yao Li

**Affiliations:** 1 Department of Orthopaedics, The Affiliated Hospital of Southwest Medical University, Luzhou City, Sichuan Province 646000, P.R. China; 2 College of Pharmacy, Southwest Medical University, Luzhou City, Sichuan Province 646000, P.R. China

**Keywords:** human fibroblast-like synoviocytes, IL-6, lncRNA DILC, rheumatoid arthritis

## Abstract

IL-6 produced by human fibroblast-like synoviocytes (HFLS) promotes rheumatoid arthritis (RA), while lncRNA DILC regulates liver cancer stem cells by inhibiting IL-6. Therefore, lncRNA DILC may participate in RA. In the present study, we found that plasma lncRNA DILC was down-regulated, while IL-6 was up-regulated in RA patients than in healthy controls. Plasma levels of lncRNA DILC and IL-6 were significantly and inversely correlated only in RA patients. Overexpression of lncRNA DILC resulted in promoted apoptosis of HFLS isolated from RA patients, while lncRNA DILC siRNA silencing played an opposite role. In addition, overexpression of lncRNA DILC also resulted in inhibited IL-6 expression in HFLS isolated from RA patients. Therefore, lncRNA DILC may participate in RA by inducing apoptosis of HFLS and down-regulating IL-6.

## Introduction

The human genome transcribes both non-coding RNAs (ncRNAs) and protein-coding mRNAs [1]. Studies on the human genome have revealed that the number of genes-transcribed ncRNAs is much bigger than the number of protein-coding genes [2]. Different from the role of mRNAs as bridges linking DNA and protein, ncRNAs directly participate in biological processes, such as the regulation of cell growth and differentiation, in the form of RNA [3]. A growing body of literature has revealed the critical functions of ncRNAs, or lncRNAs (>200 nt) in human diseases [4,5]. However, the clinical application of lncRNAs in treatment of human diseases is limited by their obscure functions.

Rheumatoid arthritis (RA) affects about 1 out of 100 people during their life time [6]. RA causes a series of clinical disorders, such as comorbidities in vascular and progressive articular destruction [7]. The continuous development of RA also increases the risk of other more severe diseases, such as cardiovascular diseases [8]. It has been reported that the altered expression pattern of lncRNAs is closely correlated with disease activity [9], indicating the involvement of lncRNAs in these diseases. LncRNA DILC regulates liver cancer stem cells by inhibiting IL-6 [11], which contribute to the development of RA [12], indicating the potential involvement of lncRNA DILC in RA. Our preliminary deep sequencing data also revealed the down-regulated expression of DILC in RA patients (data not shown). Therefore, the present study was carried out to explore the functions of lncRNA DILC in RA.

## Materials and methods

### Research subjects

A total of 75 patients with RA (patient group) and 66 healthy volunteers (control group) were enrolled in Southwest Medical University. Those participants were admitted by Southwest Medical University from March 2016 to August 2018. Patients’ inclusion criteria: (1) RA patients diagnosed in Southwest Medical University; (2) patients with complete medical history over the past 5 years; (3) patients understood the experimental principle and were willing to participate. Patients’ exclusion criteria: (1) RA patients who were treated within 3 months before admission; (2) multiple diseases were diagnosed. The 75 RA patients included 40 males and 35 females, and mean age was 49.4 ± 5.1 years. DAS28 score (disease activity) ranged from 3.99 to 5.57 (mean 4.77 ± 0.92). The 66 healthy volunteers included 36 males and 30 females, and the mean age was 49.8 ± 5.8 years. All participants signed informed consent. Ethics Committee of Southwest Medical University approved the present study.

### Plasma and human fibroblast-like synoviocyte preparations

Blood (5 ml) was extracted from the elbow vein of each participant under fasting conditions before therapies. Blood was extracted from patients before any treatment. Plasma samples were prepared through conventional method.

According to the methods described by Lee *et al.* [13], human fibroblast-like synoviocytes (HFLSs) were isolated and cultivated. HFLSs at passages 5–7 were collected for subsequent experiments.

### Real-time quantitative PCR (RT-qPCR)

Plasma samples or HFLSs were directed mixed with RNAzol reagent (Sigma-Aldrich, St. Louis, MO, U.S.A.) to extract total RNAs. RevertAid RT Reverse Transcription Kit (Thermo Fisher Scientific) was used to perform reverse transcription to synthesize cDNAs. Real-time quantitative PCR (RT-qPCR) was performed to detect the expression of lncRNA DILC and IL-6 mRNA with all PCR reaction systems prepared using Luna® Universal One-Step RT-qPCR Kit (NEB, Ipswich, MA, U.S.A.). Primers of lncRNA DILC, IL-6, and endogenous control GAPDH were designed and synthesized by GenePharma (Shanghai, China). LncRNA DILC and IL-6 mRNA expression was normalized to GAPDH based on 2^−ΔΔC^_T_ method.

### Enzyme-linked immunosorbent assay (ELISA)

Human IL-6 Quantikine ELISA Kit (D6050, R&D Systems) was used to measure plasma levels of IL-6. IL-17 levels were normalized to pg/ml before subsequent analysis.

### Cell transfection

Vectors expressing lncRNA DILC was constructed by inserting lncRNA DILC genome DNA into pCI mammalian expression vector, which was done by Sangon (Shanghai, China). LncRNA DILC siRNA and negative control siRNA were also designed and synthesized by Sangon (Shanghai, China). Lipofectamine 2000 reagent (Invitrogen, Thermo Fisher Scientific, Inc.) was used to perform all cell transfections with 10 nM vectors. Cells with no transfections were control cells. Negative controls cells were cells transfected with empty vectors or negative control siRNAs. Cells were collected 24 h after transfection before subsequent experiments.

### Cell apoptosis assay

Cell apoptosis assays were performed using cell collected at 24 h after transfection. Briefly, single cell suspensions (3 × 10^4^ cells/ml) were prepared using serum-free cell culture medium. Cells were transferred to a 6-well plate with 2 ml cell suspensions per well. Cells were cultivated for 48 h to allow cell apoptosis. After digestion with 0.25% trypsin, cells were stained with V-FITC (Dojindo, Japan) and propidium iodide (PI) (Dojindo, Japan). Finally, flow cytometry was performed to detect apoptotic cells.

### Total protein extraction and Western blotting

Total Protein Extraction Kit (NBP2-37853, Novus Biologicals) was used to extract total proteins from HFLSs at 24 h after the transfection of lncRNA DILC expression vectors. Protein concentrations were measured by BCA method, followed by electrophoresis performed using 10% SDS–PAGE gel. After gel transfer to PVDF membranes, membranes were incubated in 5% non-fat milk for 2 h at 25°C for blocking. After that, membranes were first incubated with primary antibodies of rabbit anti-human IL-6 (ab6672, 1:1000, Abcam) and GAPDH (ab9485, 1: 1000, Abcam), and secondary antibody of goat anti-rabbit IgG-HRP (1:1000, MBS435036, MyBioSource). Signals were developed using ECL (Sigma-Aldrich, U.S.A.) and normalized using Image J v1.47 software.

### Statistical analysis

Mean ± standard deviation was used to represent the data from three biological replicates. GraphPad Prism 6 software was used to perform all statistical analyses. Comparisons between plasma levels of lncRNA DILC and IL-6 between RA patients and healthy controls were performed by unpaired *t* test. Comparisons of cell apoptosis and IL-6 expression among different transfection groups were performed by one-way ANOVA and Tukey test. Pearson’s correlation coefficient was used to analyze the correlations between plasma levels of lncRNA DILC and IL-6. Differences were statistically significant at *P*<0.05.

## Results

### Plasma lncRNA DILC and IL-6 showed opposite expression pattern in RA patients

**Figure 1:**
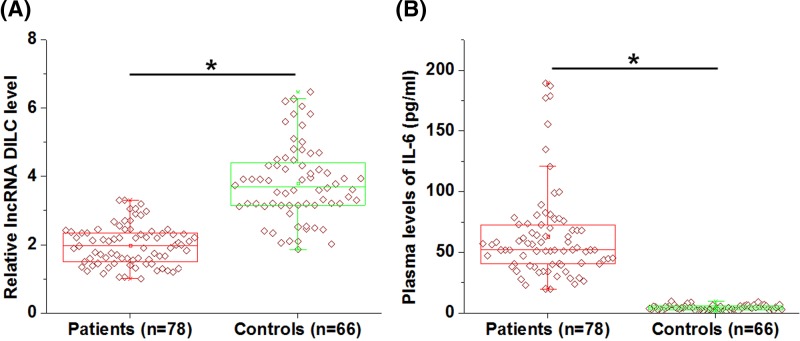
Plasma lncRNA DILC and IL-6 showed opposite expression pattern in RA patients Data of RT-qPCR and ELISA showed that plasma lncRNA DILC was significantly down-regulated (**A**), while IL-6 was up-regulated (**B**) in RA patients than in healthy controls (*P*<0.05).

Plasma levels of lncRNA DILC and IL-6 in 78 RA patients and 66 healthy volunteers were measured by RT-qPCR and ELISA, respectively. It was observed that plasma lncRNA DILC was significantly down-regulated (Figure 1A), while IL-6 was up-regulated (Figure 1B) in RA patients than in healthy controls (*P*<0.05).

### Plasma levels of lncRNA DILC and IL-6 were significantly and inversely correlated only in RA patients

**Figure 2:**
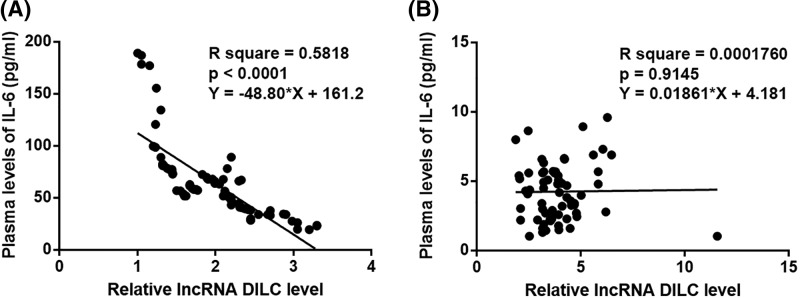
Plasma levels of lncRNA DILC and IL-6 were significantly and inversely correlated only in RA patients Pearson’s correlation coefficient analysis showed that plasma levels of lncRNA DILC and IL-6 were significantly and inversely correlated in RA patients (**A**), but not in healthy controls (**B**).

Pearson’s correlation coefficient was used to analyze the correlations between plasma levels of lncRNA DILC and IL-6. A significant and inverse correlation between plasma levels of lncRNA DILC and IL-6 was observed in RA patients (Figure 2A). However, the correlation between plasma levels of lncRNA DILC and IL-6 was not significant in healthy controls (Figure 2B).

### LncRNA DILC positively regulated apoptosis of HFLS isolated from RA patients

**Figure 3:**
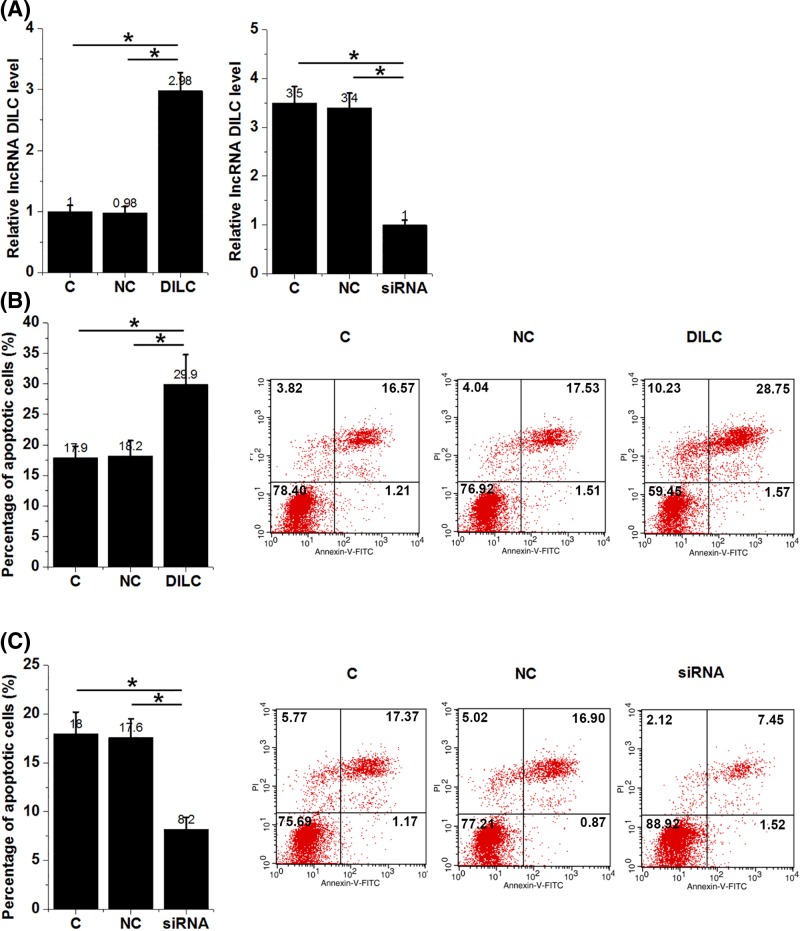
LncRNA DILC regulated apoptosis of HFLS isolated from RA patients Overexpression and knockdown of lncRNA DILC were reached at 24 h after transfection (**A**). Overexpression of lncRNA DILC led to increased (**B**), while lncRNA DILC knockdown led to decreased (**C**) apoptosis of HFLS isolated from RA patients (^*^P<0.05).

Cell apoptosis assay was performed to detect cell apoptosis after the transfection of lncRNA DILC overexpression vectors or siRNAs. As shown in Figure 3A, overexpression and knockdown of lncRNA DILC were reached at 24 h after transfection (Figure 3A, *P*<0.05). Compared with control (C) and negative control (NC) groups, overexpression of lncRNA DILC led to increased apoptosis (Figure 3B, *P*<0.05), while lncRNA DILC knockdown led to decreased apoptosis (Figure 3C, *P*<0.05) of HFLS isolated from RA patients.

### Overexpression of lncRNA DILC led to decreased IL-6 protein level in HFLS isolated from RA patients

**Figure 4:**
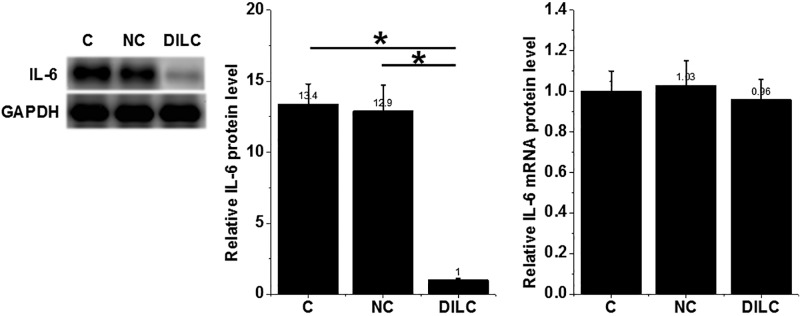
Overexpression of lncRNA DILC led to decreased IL-6 protein level in HFLS isolated from RA patients Overexpression of lncRNA DILC led to decreased IL-6 protein level in HFLS isolated from RA patients (^*^P<0.05), but failed to significantly affect IL-6 expression at mRNA level.

Compared with control (C) and negative control (NC) groups, overexpression of lncRNA DILC led to decreased IL-6 protein level in HFLS isolated from RA patients (*P*<0.05) (Figure 4). However, overexpression of lncRNA DILC failed to significantly affect IL-6 expression at mRNA level.

## Discussion

The treatment of RA is challenged by the unclear pathogenesis. The key finding of the present study is that lncRNA DILC is likely an inhibitor of RA, and the actions of lncRNA DILC in RA is likely mediated by the down-regulation of IL-6 and promoted apoptosis of HFLS.

RA is a chronic inflammatory disease that is characterized by the accelerated production of pro-inflammatory factors [13]. The increased production of IL-6 by HFLS in RA patients mediates inflammatory responses and aggregates disease conditions [11]. Therefore, inhibition of pro-inflammatory factors including IL-6 production is considered as a promising therapeutic target for RA [14–16]. Our study also observed the significantly up-regulated plasma levels of IL-6 in RA patients than in healthy controls. In some extreme cases, plasma levels of IL-6 were more than 30 times higher than the median value in healthy controls, indicating the existence of severe inflammatory responses in RA patients.

HFLS is a major contributor in the development of RA [17,18]. The activation of HFLS in RA patients secretes various pro-inflammatory factors, such as IL-6 and IL-8, to promote the development of RA [11]. Therefore, inhibition of HFLS is critical for the recovery of RA patients [19]. In the present study we first reported the down-regulation of lncRNA DILC in RA patients. Interestingly, our study also proved that lncRNA DILC promoted the apoptosis of HFLS, suggesting that the overexpression of lncRNA DILC may serve as a therapeutic target for RA.

It is known that the expression of IL-6 in human diseases can be regulated by certain lncRNAs [20]. A recent study reported that lncRNA DILC can bind to the promoter region of IL-6 to inhibit its expression [10]. Interesting, our study did not observed significantly reduced IL-6 mRNA in HFLS after lncRNA DILC overexpression. In contrast, lncRNA DILC overexpression led to significantly reduced IL-6 protein levels in HFLS. To observe the potential changes in expression levels of IL-6 mRNA at early time points after DILC overexpression, we further detected the expression of IL-6 mRNA at 2, 4, and 8 h after transfection, but still no significant changes were observed (data not shown). It is known that lncRNAs may regulate gene expression at post-transcriptional, translational, and epigenetic levels [4,5]. Therefore, DILC may regulate IL-6 at translational level or DILC may regulate the levels of IL-6 in HFLS by affecting protein degradation or accumulation. In addition, DILC and IL-6 were significantly and inversely correlated only in RA patients, but not in healthy controls, indicating the possible existence of RA-related pathological factors mediating the interaction between DILC and IL-6.

It is worth nothing that we did not observed significant effects of IL-16 on cell apoptosis. In addition, no significant changes in expression levels of several classic apoptotic factors, such as caspase-9, caspase-3, p53, Bax, and Bcl2 [21–23] were observed after DIHC overexpression (data not shown). Therefore, the mechanism of the regulated apoptosis of HFLS by DILC is still unknown. Our future studies will try to consider other apoptosis-related factors. In conclusion, lncRNA DILC was down-regulated in RA and overexpression of lncRNA DILC may improve RA by down-regulating IL-6 and inhibiting the apoptosis of HFLS.

## Abbreviations

HFLS: human fibroblast-like synoviocyte
ncRNA: non-coding RNA
RA: rheumatoid arthritis
RT-qPCR: real-time quantitative PCR
